# Motivations and barriers to engaging in peer review: a qualitative study

**DOI:** 10.1186/s41073-026-00208-z

**Published:** 2026-06-18

**Authors:** Júlio Belo Fernandes, Ana Silva Almeida, Faiza Magsi, Ana Catarina Maia, Sónia Fernandes

**Affiliations:** 1 Egas Moniz Center of Interdisciplinary Research (CiiEM), Egas Moniz School of Health & Science, Monte da Caparica, Almada, Portugal; 2 Nurs* Lab, Monte da Caparica, Almada, Portugal; 3 Arrábida Local Health Unit, Hospital de São Bernardo, Setúbal, Portugal; 4 University of Nevada, Las Vegas, USA; 5 Health Sciences Research Unit: Nursing (UICISA: E), Nursing School of Coimbra (ESEnfC), Coimbra, Portugal

**Keywords:** Peer review, Reviewer engagement, Motivations, Barriers, Challenges

## Abstract

**Background:**

Peer review is a central mechanism of scientific communication. However, despite its critical role in safeguarding research quality and integrity, there is limited evidence on how reviewers themselves perceive the factors that motivate or hinder their engagement. This study explored reviewers' perceptions of the motivations and barriers shaping participation in peer review.

**Methods:**

A qualitative, exploratory-descriptive design was adopted. Semi-structured interviews were conducted between June and September 2025 with participants holding an academic title in health sciences who had completed at least one peer review for a scientific journal. Participants were recruited through purposive sampling. Interviews were audio-recorded, transcribed verbatim, and analysed inductively using thematic analysis following Braun et al.’s framework. Data collection and analysis proceeded iteratively until thematic saturation was reached.

**Results:**

Twenty-seven academics from seven health science disciplines participated in the study. Participants were predominantly female (63%), with similar proportions holding doctoral (52%) and master's degrees (48%), and peer-review activity in the previous 12 months ranging from 1 to more than 10 reviews. Findings were organised into two domains, motivations and barriers, comprising ten themes. Motivations included contribution to science, scientific development, career development, personal satisfaction, and financial incentives. Barriers included high workload, lack of recognition and incentives, perceived competence, research integrity, and editorial shortcomings.

**Conclusion:**

Reviewer engagement appears to be a negotiated process in which academics weigh motivations against barriers when deciding whether to participate in peer review. These trade-offs shape decisions to accept or decline review invitations. Strengthening recognition, transparency, and editorial support may help sustain reviewer participation in the health sciences.

**Supplementary Information:**

The online version contains supplementary material available at 10.1186/s41073-026-00208-z.

## Introduction

Peer review has long been embedded in the practices of scientific communication, emerging as a foundational mechanism for evaluating scholarly work and supporting the credibility of academic journals [1]. As research output expanded and scientific disciplines became increasingly specialised, peer review evolved into a more structured and systematic process [2, 3]. Regardless of the model adopted, its central purpose remains consistent: to safeguard the quality, validity, originality, and integrity of scientific research [4].

The role of the peer reviewer involves the critical evaluation of submitted manuscripts and the provision of constructive, impartial, and transparent feedback to both authors and editors. This responsibility presupposes not only subject-matter expertise but also adherence to principles of fairness, professionalism, and ethical conduct [5]. By undertaking the reviewer role, academics implicitly commit to upholding the core values of scientific practice, contributing to research conducted responsibly and in ways that protect participants and strengthen the evidence base [6]. In this sense, peer review extends beyond journal publishing to function as a central pillar of scientific practice, with the potential to help advance knowledge and strengthen the quality of research dissemination [5]. By exposing manuscripts to scrutiny by disciplinary experts, peer review contributes to methodological robustness, conceptual clarity, and ethical accountability, thereby enhancing the trustworthiness of published findings [7]. Despite this widely acknowledged role, the contemporary peer review system is increasingly characterised as vulnerable and under pressure, largely dependent on the voluntary and often unrecognised contributions of academic reviewers [8, 9].

Concerns regarding the sustainability of peer review have intensified alongside rising manuscript submission rates, expanding editorial responsibilities, and ongoing productivity pressures within academia [8, 10, 11]. These dynamics have led to growing demands on reviewers’ time and cognitive resources, contributing to overload, fatigue, and, in some cases, reduced willingness to participate. The magnitude of this contribution is substantial: estimates suggest that a relatively small proportion of highly active reviewers accounts for a disproportionate share of review activity, investing vast amounts of unpaid labour to sustain the system [8, 10, 11].

The demands of peer review extend across all actors involved in the publication process. Authors are required to engage critically with feedback and revise or defend their work; editors must coordinate reviews and make complex decisions regarding colleagues’ manuscripts; and reviewers must dedicate time to careful and thoughtful evaluation, often in competition with other academic responsibilities and without financial compensation [12, 13]. A particularly challenging and rate-limiting step in this process is the identification and recruitment of suitable reviewers. Repeated refusals to review are common and can significantly delay editorial workflows, increase the burden on editors, and affect the overall efficiency of the review process [10, 11, 14].

Despite growing concern about the sustainability of peer review, the current evidence base remains fragmented. Much of the literature has examined peer review from editorial, bibliometric, or policy perspectives, often relying on quantitative surveys or large-scale system analyses. Comparatively less attention has been paid to how reviewers themselves interpret and negotiate the conditions that enable or constrain their participation in everyday academic practice. This leaves an important experiential and interpretive gap in understanding reviewer engagement.

Recent meta-science scholarship has also proposed multiple systemic responses to the pressures facing peer review, including open peer review models, distributed or community-based reviewing, and incentive restructuring. For example, Waltman et al. describe four competing “schools of thought” for improving peer review, ranging from incremental optimisation of the current system to more transformative redesigns [15].

Complementary perspectives have raised concerns about bias, fairness, and the need to reimagine peer review processes to ensure equity and transparency [16, 17]. In parallel, ongoing debate continues regarding the potential role, benefits, and risks of financially compensating reviewers [18]. While these contributions provide important macro-level insights, they offer limited qualitative understanding of how reviewers themselves make sense of these evolving expectations and reforms.

Within the health sciences in particular, this gap is especially relevant. Peer review practices and expectations can vary across disciplinary contexts, reflecting differences in publication cultures, methodological traditions, and professional norms, as well as different perspectives on how peer review should function within the scholarly system [15, 19]. Health-related fields are characterised by high publication volume, strong clinical–academic role overlap, and increasing methodological complexity, all of which may shape reviewers’ experiences in distinctive ways [20, 21].

Accordingly, examining reviewers within the health sciences offers a contextually grounded opportunity to better understand how disciplinary conditions may influence engagement in peer review. Understanding these perspectives is important because workload configurations, professional norms, and accountability expectations in health disciplines may intensify both motivational drivers and participation barriers.

In light of these challenges, a clearer understanding of why academics in the health sciences choose to engage in peer review, and what hinders or enables their participation, has become increasingly important. However, there remains limited insight into how reviewers themselves make sense of these factors and negotiate them in everyday academic practice. To address this gap, the present study explores reviewers’ perceptions of the motivations and barriers shaping engagement in the peer review process.

## Methods

### Study design

A qualitative, exploratory-descriptive design was adopted to explore reviewers’ perceptions of the motivations and barriers to engaging in the peer review process. The Consolidated Criteria for Reporting Qualitative Research (COREQ) checklist was used to guide the reporting of this study and to enhance the transparency, completeness, and methodological rigour of the manuscript [22].

The study was conducted in Portugal, and ethics approval was obtained from the Egas Moniz Ethics Committee (Reference Number: 1591).

### Participants and recruitment

Participants were recruited using purposive sampling to ensure relevance and information-rich accounts. Eligibility criteria were: (i) holding an academic title in health sciences, and (ii) having completed at least one peer review for a scientific journal.

Recruitment was conducted in two phases. First, the study was disseminated on social media platforms to raise awareness and enable the pre-registration of potential participants. Interested individuals completed a brief registration survey designed to support purposive sampling, including items on academic title, years of academic experience, and recency of involvement in peer review.

During sampling, demographic and professional characteristics collected at pre-registration were reviewed iteratively to support variation across career stages, academic roles, and levels of peer-review activity, thereby enhancing diversity of perspectives within the sample.

**Fig. 1:**
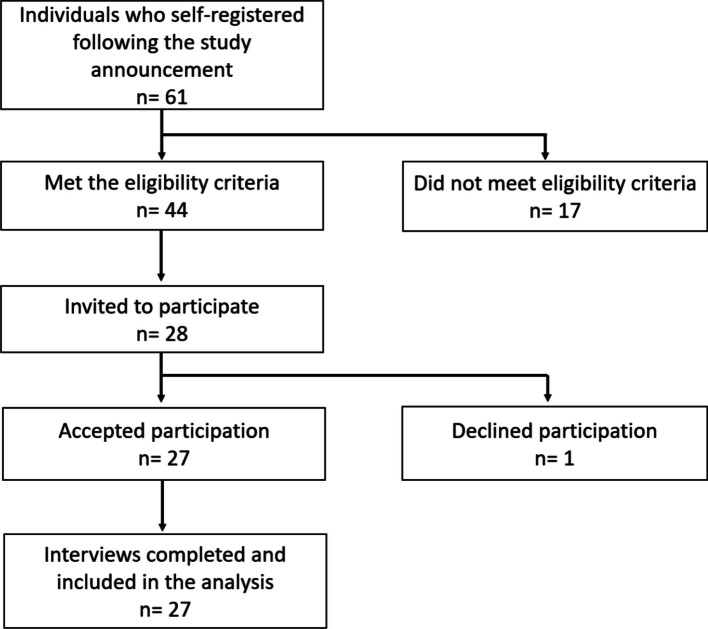
Study participant flow chart

In the second phase, individuals meeting the eligibility criteria were contacted by email. The recruitment process is illustrated in Fig. 1. The invitation included detailed written information about the study aims and procedures, allowing potential participants time to review the information and reflect on the topic before deciding whether to participate. A reminder email was sent one week after the initial invitation to individuals who had not yet responded.

### Data collection procedures

Interviews were conducted between June and September 2025 at times convenient to participants using Microsoft Teams through the institutional license provided by Egas Moniz School of Health and Science (Microsoft Corporation, Redmond, WA, USA). All interviews were carried out in Portuguese, the participants’ native language. Only the interviewer and the participant were present during each interview.

All interviews were conducted by the lead researcher (JBF), a professor with a PhD in Psychology, training and experience in qualitative research, and established interviewing skills. The interviewer had no prior relationship with the participants.

Interviews proceeded only after participants had received study information and provided informed consent. Interviews proceeded only after participants had received study information and provided informed consent. A total of 27 interviews were conducted. Although 44 participants met the eligibility criteria, 28 were invited to participate and initially agreed to take part. One participant subsequently withdrew due to personal scheduling constraints. Interviews were digitally audio-recorded and transcribed using the automated transcription function of Microsoft Teams. The transcripts were subsequently reviewed and manually verified against the original audio recordings by the research team to ensure accuracy and completeness.

Data were collected using a semi-structured interview guide designed by the authors. The guide included (i) questions capturing participant characteristics and descriptive peer review behaviour, and (ii) open-ended prompts exploring perceptions of motivations and barriers influencing engagement in peer review. The guide was pilot tested with individuals who met the eligibility criteria to assess clarity, flow, and relevance. Minor refinements were made to improve wording and ensure questions were sufficiently clear, comprehensive, and non-leading, minimising the risk of ambiguity or equivocal interpretation. The interviews had a mean duration of 36.8 min. Field notes were not taken during or after the interviews, and repeat interviews were not deemed necessary.

### Sample size and saturation

In line with qualitative research principles, sampling was guided by the richness and relevance of participants’ accounts rather than by a predetermined target number [23, 24]. No fixed sample size was established in advance. Data collection and analysis proceeded iteratively, and recruitment continued until thematic saturation was reached, defined as the point at which no substantive new information or themes emerged [25]. Saturation was monitored throughout concurrent analysis and was operationalised as the absence of new insights across three consecutive interviews.

### Data analysis

Data were analysed using reflexive thematic analysis, following Braun et al. [26] six-phase approach. Analysis was inductive and data driven. Two members of the research team were involved in coding.

Transcripts were read repeatedly for familiarisation with the data, after which initial codes were generated across the dataset. Codes were then collated into candidate themes, which were subsequently reviewed and refined in relation to both the coded extracts and the full dataset. Themes were clearly defined and named to capture their analytic focus, and the final phase involved the production of the report through the selection of illustrative quotations and the development of the analytic narrative. QDA Miner Lite qualitative data analysis software was used to support data management, coding, and organisation.

Selected quotations included in this manuscript were translated into English by the research team using a back-translation procedure. Initial translations were produced by bilingual team member and independently back-translated into Portuguese by a second bilingual reviewer. Any discrepancies were reviewed and resolved by a third independent bilingual reviewer to ensure conceptual and semantic equivalence.

### Trustworthiness and reflexivity

The study was conducted by researchers with experience in qualitative research and scholarly publishing. The research team included academics familiar with the peer-review process, both as authors and reviewers. This background facilitated a contextual understanding of participants' accounts, while reflexive strategies were used throughout the study to minimise bias and avoid presuppositions that might influence data interpretation. Reflexive discussions were held within the team during data analysis to examine interpretations and enhance analytical transparency critically.

Trustworthiness was ensured by applying strategies consistent with credibility, dependability, confirmability, and transferability [27]. Credibility was enhanced through repeated engagement with the dataset (familiarisation and iterative re-reading), reflexive discussions within the research team during coding and theme development, and the use of open, non-leading questions to support authentic accounts and reduce social desirability influences. Dependability was supported by maintaining an audit trail documenting methodological and analytic decisions throughout the study and by regular team meetings to discuss coding consistency and refine themes. Confirmability was strengthened by ensuring that interpretations were demonstrably grounded in the data through iterative checking of themes against transcripts and by documenting key analytic decisions. Transferability was supported by a clear description of the study context, participant characteristics, and analytic approach, enabling readers to assess its relevance to other settings.

## Results

A total of 27 interviews were conducted. Participants were predominantly female (63%), with similar proportions holding doctoral (52%) and master's degrees (48%). Academic titles and experience were heterogeneous, and peer-review activity in the previous 12 months ranged from 1–2 to more than 10 (Table 1).

**Table 1:** Participants’ characteristics

Variable	Category	n	%
**Sex**	Female	17	63
	Male	10	37
**Academic degree**	PhD	14	52
	MSc	13	48
**Discipline**	Dentistry	3	11
	Medicine	4	15
	Nursing	5	18.5
	Nutrition/dietetics	3	11
	Pharmacy	4	15
	Physiotherapy	3	11
	Psychology	5	18.5
**Academic title**	Lecturer/Adjunct	6	22
	Assistant Professor	7	26
	Associate Professor	6	22
	Full Professor	2	8
	Researcher (non-faculty)	6	22
**Years of academic experience**	0–5 years	8	30
	6–10 years	4	15
	11–15 years	6	22
	16–20 years	4	15
	> 20 years	5	18
**Peer review activity**	1–2 reviews in the last 12 months	8	30
	3–5 reviews in the last 12 months	9	33
	6–10 reviews in the last 12 months	6	22
	> 10 reviews in the last 12 months	4	15

The analysis revealed a nuanced set of motivations and barriers shaping academics' engagement in the peer review process. These findings are organised into two overarching domains: motivations for participating in peer review and barriers to engagement, each comprising several interrelated themes and subthemes (Table 2).

**Table 2:** Motivations and barriers for participating in peer review

Domain	Themes	Subthemes
Motivations	Contribution to science	Ensuring scientific quality and rigor
		Sustainability of the peer review system
	Scientific development	Continuous learning
		Privileged access to knowledge
		Conceptual depth
		Training offered by publishers
	Career development	Career progression
		Reputation building
		Professional networking
	Personal satisfaction	-
	Financial incentives	-
Barriers	High workload	Multiple academic and clinical responsibilities
		Short editorial deadlines
		Reviewer fatigue
	Lack of recognition and incentives	Lack of institutional recognition
		Low perceived return
		Insufficient editorial incentives
	Perceived competence	Methodological or statistical insecurity
		Lack of peer review training
	Research integrity	Predatory journals
		Unclear journal legitimacy
		Misuse of reviewer identity
	Editorial shortcomings	Low-quality or immature manuscripts
		Vague editorial guidelines
		Lack of transparency in editorial processes

### Motivations for participating in peer review

Participants described peer review as a meaningful scholarly activity grounded in both collective responsibility and individual professional development. Motivations extended beyond altruistic service to include learning opportunities, career-related benefits, and the reinforcement of academic identity.

#### Contribution to science

Participants perceived peer review as a key mechanism for safeguarding scientific quality, methodological rigour, and the credibility of published research. Ensuring the robustness and trustworthiness of the evidence base was framed as a core professional duty. This motivation was tied to a strong sense of responsibility for the sustainability of the peer-review system, often articulated through reciprocity: reviewing others' work in response to having one's own manuscripts reviewed. Accordingly, participants emphasised that the continued functioning of peer review depends on collective engagement and shared commitment across the scientific community.

Peer review is one of the few safeguards we have to ensure that what is published is trustworthy. I see it as a responsibility. Others review my work, so I feel committed to reviewing theirs.

#### Scientific development

Peer review engagement was recognised as an opportunity for continuous professional learning. Participants valued exposure to emerging topics and diverse theoretical perspectives, often describing peer review as an informal yet powerful learning space.

Reviewing allows me to stay intellectually active. I am exposed to new methods, different approaches, and perspectives that I might not encounter otherwise.

Participants also highlighted the motivational role of privileged access to new knowledge prior to publication, alongside the opportunity to deepen conceptual and theoretical thinking.

Engaging in peer review encourages more critical reflection on concepts and theory, which in turn enhances the quality of my own research.

Additionally, training opportunities offered by journals and publishers were perceived as additional incentives, particularly when they enhanced methodological confidence and academic writing skills.

#### Career development

Several motivations were linked to professional advancement. Peer review was perceived as contributing to career progression, particularly when acknowledged in promotion processes or institutional evaluations. Participants also reported that engaging in peer review enhanced their reputation, reinforcing their identity as subject-matter experts and increasing international visibility. Professional networking was also identified as a motivating factor, especially when sustained reviewing led to closer engagement with editors and editorial boards.

Being invited to review signals recognition of expertise. It helps build credibility and opens doors, particularly in terms of relationships with editors and editorial boards.

#### Personal satisfaction

Peer review was described as a source of personal fulfilment, characterised by enjoyment, a sense of service, and satisfaction derived from contributing to science in less visible ways. Participants highlighted the rewarding nature of supporting the scientific community "behind the scenes".

There is a sense of satisfaction in knowing that you are contributing behind the scenes. It is part of how I understand my role as an academic.

#### Financial incentives

Participants also acknowledged financial incentives, such as vouchers or discounts on article processing charges, as pragmatic motivators. In the context of limited funding, these benefits were perceived as enabling the dissemination of their own work in open access.

The vouchers are not the main reason I do a review, but they do help, especially when funding is limited.

### Barriers to participation in peer review

Despite recognising the value of peer review, participants reported multiple barriers that constrained their capacity and willingness to engage. These barriers were largely structural and organisational, reflecting broader conditions of academic work.

#### High workload

Participants identified day-to-day workload as a key barrier to engaging in peer review. Balancing multiple academic and clinical responsibilities constrained the time and cognitive space needed to produce careful, high-quality reviews.

Between teaching, supervising students, writing my own papers, grants, and administrative tasks, peer review often becomes something I do late at night or during weekends.

In addition to managing heavy workloads, participants perceived short editorial deadlines as unrealistic, undermining the time needed for careful reading, reflection, and the provision of constructive feedback. Repeated invitations, often directed to the same limited pool of reviewers, were described as further intensifying overload and exacerbating reviewer fatigue.

Some deadlines simply do not allow for careful reading and reflection. It feels incompatible with the level of rigour expected from a good review.

#### Lack of recognition and incentives

Participants highlighted a persistent lack of institutional recognition for peer review activities. The effort invested was often seen as disproportionate to the low perceived return, given limited visibility, minimal impact on performance evaluations, and scarce consideration in funding decisions. Inconsistent or absent editorial incentives further reinforced perceptions that peer review labour was undervalued. Participants referred to variability in journal practices, including the absence of formal acknowledgement, limited feedback on the quality of their reviews, and the irregular provision of tangible benefits such as vouchers or discounts on article processing charges. This inconsistency contributed to a broader perception that reviewers’ contributions were insufficiently recognised across both institutional and editorial contexts.

Peer review takes time and intellectual effort, yet it is unpaid labour for journals that benefit from it, and it is rarely valued by institutions in promotion, grant decisions, or workload models. It feels invisible.

#### Perceived competence

Perceived competence-related barriers also shaped participation. Participants described methodological or statistical insecurity, particularly when manuscripts relied on unfamiliar techniques, which made them hesitant to accept review invitations. The lack of formal training in peer review further intensified uncertainty about expectations, standards, and assessment criteria.

There are times when I hesitate to accept a review because the methods are highly specialised, and I am not confident I can add real value.

#### Research integrity concerns

Concerns about research integrity affected reviewers’ willingness to accept invitations. Fear of engaging with predatory journals, uncertainty about journal legitimacy, and difficulties distinguishing credible outlets from fraudulent ones were recurrently mentioned. Participants also raised apprehensions about the misuse of reviewer identity. This included concerns that their names or reviewer status could be used without explicit consent, uncertainty about how their reviews might be stored or shared, and fears of unethical exploitation of reviewer labour within opaque editorial systems. Such concerns contributed to hesitancy in accepting review invitations, particularly when journal credibility was uncertain.

It is not always easy to distinguish legitimate journals from predatory ones. When in doubt, I prefer to decline.

#### Editorial shortcomings

Participants also highlighted barriers related to manuscript quality and editorial processes. Low-quality or immature submissions, particularly those that were poorly prepared, outside the journal's scope, or characterised by high levels of textual similarity, were perceived as time-consuming and demotivating. Vague or inconsistent editorial guidelines further created uncertainty regarding the expected depth and focus of reviews. In addition, the lack of feedback to reviewers, such as not being informed of editorial decisions or how their comments were used, was described as frustrating and diminished motivation to accept future review invitations.

"Reviewing manuscripts that are clearly immature or outside the journal's scope feels like a misuse of time … when editorial decisions are unclear, and communication with reviewers is poor, it raises questions about how reviewers' work is actually used.”

## Discussion

The study findings indicate that participation is shaped by ongoing tensions between a strong responsibility towards science and increasingly demanding conditions of contemporary academic work. Importantly, participants’ accounts suggested that single factors rarely drove decisions to accept or decline review invitations; instead, reviewers described weighing professional commitment against competing time pressures, perceived recognition, and confidence in the review context.

Reviewer engagement was strongly anchored in normative expectations of scholarly contribution and community responsibility, reinforcing the notion of peer review as a form of scholarly citizenship. While this moral framing may help sustain participation in the absence of formal rewards, it simultaneously exposes the fragility of a system that relies heavily on goodwill and reciprocity [28]. Such commitment, although persistent, may be insufficient to counterbalance mounting structural pressures over time [8, 9, 12].

The findings highlight peer review as an important space for learning and intellectual development. This reinforces the view of peer review not only as a gatekeeping mechanism but also as a pedagogical practice embedded within academic work [29]. At the same time, the developmental benefits associated with reviewing raise questions about equity, as those with lower methodological confidence or limited access to training may be less likely to participate, potentially reinforcing existing hierarchies within academia.

Career-related motivations further illustrate the ambivalence surrounding peer review. While participants acknowledged reputational and networking benefits, these were perceived as uncertain and inconsistently recognised. This ambiguity reflects a misalignment between the symbolic value of peer review within scholarly culture and its marginal status in institutional reward systems. The findings suggest that peer review occupies a paradoxical position: widely described as essential to science, yet weakly embedded in formal mechanisms of academic recognition [30]. Taken together, these patterns portray reviewer engagement in the health sciences as a negotiated practice shaped by competing professional, institutional, and systemic demands. This balancing process underscores the relational nature of reviewer engagement, in which workload constraints, limited recognition, or concerns about journal credibility often tempered motivating factors.

The identification of personal satisfaction as a distinct motivator is particularly revealing. This finding nuances dominant narratives that frame academic motivation primarily in terms of productivity or career advancement, instead foregrounding the affective dimensions of scholarly work, an aspect also identified in previous research [3]. However, reliance on personal fulfilment as a sustaining force risks normalising unpaid and invisible labour, potentially masking more profound structural inequities.

At the same time, the reported barriers underscore the limits of individual motivation when confronted with organisational constraints. Workload pressures, unrealistic deadlines, and reviewer fatigue were experienced not as isolated inconveniences but as manifestations of broader transformations in academic labour. These pressures force reviewers to position peer review in competition with activities that are more directly rewarded, such as publishing and grant acquisition. These dynamics may be particularly pronounced in the health sciences, where academic roles frequently intersect with clinical and teaching responsibilities. If left unaddressed, such conditions may affect both the sustainability and quality of peer review [8, 9, 12]. Participants’ narratives indicated that these pressures did not simply deter engagement; rather, reviewers described making pragmatic judgements about when the perceived value of reviewing outweighed competing demands.

The lack of recognition and incentives further exacerbates this tension. While financial incentives were seen as helpful, they were insufficient to address the underlying issue of symbolic and structural undervaluation. This supports arguments that improving reviewer engagement requires systemic recognition rather than isolated compensatory measures [31–33].

Concerns related to competence and research integrity point to the role of trust, both self-trust and system trust, in shaping engagement. Together, these findings suggest that reviewer engagement depends not only on individual capability but also on the perceived integrity and fairness of the systems in which reviewers are asked to participate.

Finally, issues related to manuscript quality and editorial communication highlight how editorial practices actively shape reviewers' experiences. Poor-quality submissions, vague guidance, and a lack of feedback signal to reviewers that their efforts may not be valued or used effectively. These signals appear to influence decisions about future engagement, reinforcing the idea that reviewer motivation is relational and context-dependent rather than purely individual.

### Strategies for enhancing peer review engagement

**Fig. 2:**
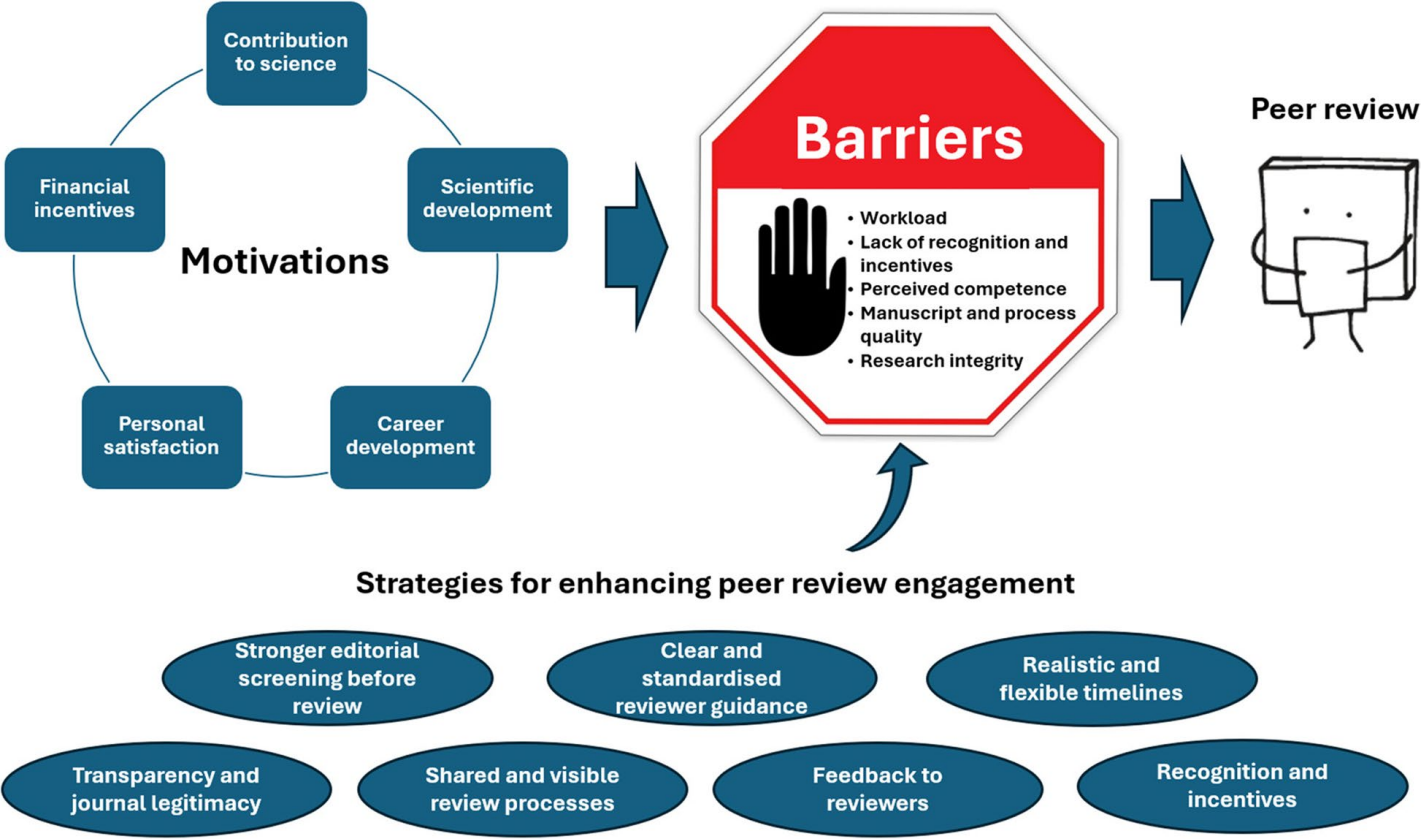
Targeted actions

Building on the tensions identified, the following strategies are proposed as practice-informed implications of the findings (Fig. 2). Importantly, several of these directions converge with ongoing developments in meta-research and open science. In particular, participants’ emphasis on transparency, feedback, and meaningful recognition aligns with broader shifts toward open peer review models, shared and visible review processes, and community-based evaluation approaches increasingly adopted by publishers and scholarly platforms. These developments resonate with international initiatives seeking to reform research assessment and peer review practices, including the Declaration on Research Assessment (DORA), the Leiden Manifesto, and the Coalition for Advancing Research Assessment (CoARA), which advocate for greater transparency, recognition of diverse scholarly contributions, and more responsible evaluation systems. Situating the present findings within this evolving landscape strengthens their relevance and highlights the systemic conditions under which reviewer engagement may be more sustainably supported.

First, stronger editorial screening before manuscripts are sent for peer review may help reduce unnecessary reviewer burden [34, 35]. Participants frequently described frustration with immature, out-of-scope, or low-quality submissions, which consumed time and undermined motivation. More rigorous initial triage procedures could therefore protect reviewers’ cognitive resources while signalling respect for their time and expertise.

Second, clear, standardised reviewer guidelines, including brief checklists tailored to study type, can reduce uncertainty about expectations and help reviewers focus their efforts [36]. Coupled with realistic and negotiable deadlines, these measures may alleviate time pressure and mitigate reviewer fatigue.

Third, enhancing transparency and trust in the peer review system is critical. Concerns about journal legitimacy and the ethical use of reviewer labour shaped participants’ willingness to accept invitations. Journals should include direct links to recognised institutional bodies within review invitations to support reviewers in assessing journal legitimacy. Transparency may be further strengthened by sharing elements of the review process with reviewers, such as access to other reviewers' comments and iterative, real-time exchanges, which allow reviewers to refine their assessments and better understand editorial decision-making [15, 37].

Fourth, recognition and incentives should move beyond symbolic appreciation [15, 18]. Participants described peer review as insufficiently valued within formal workload models, promotion pathways, and research assessment processes. While vouchers or article processing charge discounts can act as pragmatic facilitators, more sustainable engagement requires formal recognition of peer review within institutional workload models, promotion criteria, and performance evaluations. Beyond the institutional level, peer review activity should also be acknowledged in broader academic evaluation processes, including professional career progression frameworks and, where applicable, grant and funding assessments. At the journal level, editorial boards should make explicit the criteria used to value both the quantity and quality of reviews, including how reviewing activity contributes to invitations to join editorial boards or assume advanced reviewer roles.

Finally, capacity-building initiatives, such as short training modules, mentoring, and access to exemplary reviews, may reduce competence-related barriers and support equitable participation across career stages [38, 39].

Together, these measures can help reposition peer review as a transparent, supported, and valued scholarly activity, rather than an invisible obligation that relies primarily on individual goodwill.

### Limitations

This study has limitations that should be considered when interpreting the findings. Because participants were drawn exclusively from the health sciences, the transferability of these findings to other disciplinary contexts should be considered with caution. Peer review cultures, workload configurations, and incentive structures may differ across fields. Participants were self-selected and had prior experience with peer review, which may have introduced selection bias and favoured the inclusion of individuals with relatively higher intrinsic motivation to engage in peer review, thereby underrepresenting more disengaged or marginalised voices.

More broadly, because the data capture reviewers’ perceptions, the study cannot determine whether any of the reported barriers reflect consistent, journal-specific editorial practices or are shaped by cumulative impressions formed through isolated negative experiences; thus, the findings represent perceived patterns rather than empirically verifiable differences across journals, editorial models, or publisher groups. Finally, the study relied solely on reviewers’ accounts and did not include editor perspectives or journal-level documentation (e.g., editorial policies or decision timelines), limiting triangulation and preventing direct comparison between reviewers’ interpretations and editorial intentions or constraints.

Despite these constraints, the study provides valuable insight into how reviewers interpret editorial signals and how these interpretations shape willingness to engage in peer review.

Future research should extend these exploratory findings by examining whether similar patterns of reviewer engagement are observed across disciplinary contexts beyond the health sciences. Quantitative or mixed-methods studies could usefully test the relative influence of the factors identified in this study in shaping reviewers’ acceptance decisions. In addition, multi-perspective studies incorporating editors and less engaged reviewers would further clarify how individual and systemic factors interact to shape participation in peer review.

## Conclusion

This study provides an in-depth qualitative exploration of the motivations and barriers shaping academics’ engagement in peer review, highlighting the complex interplay between professional commitment, personal meaning, and structural conditions of contemporary academic work. Reviewers’ participation was grounded in a strong sense of responsibility towards science, reciprocity, and collective stewardship of research quality, alongside motivations related to learning, career development, and personal satisfaction. These motivations, however, were continuously negotiated against organisational pressures and systemic constraints.

The findings demonstrate that disengagement from peer review is rarely driven solely by a lack of willingness or expertise. Instead, it emerges from accumulated experiences of workload pressure, limited recognition, competence-related uncertainty, and concerns about research integrity and editorial practices. Importantly, reviewers' accounts underscore that engagement is relational and context-dependent: perceptions of journal legitimacy, editorial transparency, manuscript quality, and communication practices shape how reviewers interpret the value and impact of their contributions and influence decisions about future participation.

By foregrounding reviewers’ perspectives, this study contributes to ongoing debates about the sustainability of peer review and challenges individualised narratives that frame disengagement as a matter of personal motivation or time management. Instead, the findings point to the need for systemic and relational approaches that recognise peer review as skilled academic labour embedded within broader evaluation and reward structures.

## Supplementary Information

Additional file 1

Additional file 2

## Data Availability

The datasets used and/or analysed during the current study are available from the corresponding author on reasonable request.
