# Supplementary material for: Motivations and barriers to engaging in peer review: a qualitative study
Source: Res Integr Peer Rev. 2026 Jun 18;11:23. doi: 10.1186/s41073-026-00208-z (PMC13277020; doi:10.1186/s41073-026-00208-z)
Supplement: Supplementary file 2 — Additional file 2 [file 41073_2026_208_MOESM2_ESM.docx]

**Semi-structured interview guide**

1. When you receive an invitation to review, how do you decide whether to accept or decline?
2. What factors make you decline or hesitate to accept the invitation?
3. What motivates you to participate in the peer review process?
4. What benefits, if any, do you perceive from engaging in peer review?
5. What are the main barriers you identify to participating in the peer review process?
6. How do these barriers affect your willingness or ability to participate?
7. What costs do you associate with the peer review process?
8. Which factors facilitate your participation in peer review?
9. Is there any type of incentive that you believe could improve reviewer participation in the peer review process?
10. Do you feel that your work as a reviewer is recognised? In what ways does recognition influence your willingness to participate in future reviews?
11. How does peer review relate to your academic or professional responsibilities and professional development?

**Closing question**

Is there anything else about your experience with peer review that you would like to share?
